# Preclinical Assessment of Paclitaxel- and Trastuzumab-Delivering Magnetic Nanoparticles Fe_3_O_4_ for Treatment and Imaging of HER2-Positive Breast Cancer

**DOI:** 10.3389/fmed.2021.738775

**Published:** 2021-10-28

**Authors:** Liting Guo, Hongming Zhang, Ping Liu, Tianai Mi, Da Ha, Li Su, Lei Huang, Yan Shi, Jun Zhang

**Affiliations:** ^1^Department of Oncology, Ruijin Hospital, Shanghai Jiao Tong University School of Medicine, Shanghai, China; ^2^Department of Respiratory Medicine, Yancheng Third People's Hospital, The Affiliated Yancheng Hospital of Southeast University Medical College, Yancheng, China; ^3^Department of Oncology, Jiangsu Institute of Cancer Research, Jiangsu Cancer Hospital, Nanjing Medical University Affiliated Cancer Hospital, Nanjing, China; ^4^Lianren Digital Health Technology Company, Ltd., Shanghai, China

**Keywords:** breast cancer, magnetic nanoparticle Fe_3_O_4_, Herceptin, targeted drug delivery system, targeted therapy

## Abstract

**Objective:** The purpose of this study was to investigate the anticancer activity and the potential imaging use of the innovative combination of magnetic nanoparticles (MNPs)-Fe_3_O_4_, paclitaxel (PTX), and trastuzumab (Herceptin) in HER2-positive breast cancer.

**Methods:** MNPs-Fe_3_O_4_ was synthesized and underwent water phase transfer and hydrophobic molecular loading, and its surface was then coupled with Herceptin mono-antibody. The morphological characteristics of MNPs-Fe_3_O_4_ were observed under transmission electron microscopy (TEM). Effects of PTX-Herceptin-MNPs-Fe_3_O_4_ on breast cancer cells were evaluated using the 3-(4,5-dimethylthiazol-2-yl)-2,4-diphenyltetrazolium bromide assay and the flow cytometric apoptosis assay. To establish a xenograft model, we injected breast cancer SK-BR-3 cells into the left thighs of nude mice. We measured the effect of PTX-Herceptin-MNPs-Fe_3_O_4_ on tumor growth by measuring tumor size and calculating inhibition rate with immunohistochemistry analysis further performed, and analyzed MNPs-Fe_3_O_4_ accumulation in tumor lesions using *in vivo* magnetic resonance imaging and *in vivo* fluorescence imaging.

**Results:** Most MNPs were in spherical shape of about 10 nm in diameter observed under TEM. PTX-Herceptin-MNPs-Fe_3_O_4_ showed greater cytotoxic effects, and induced a higher apoptosis rate of SK-BR-3 cells than all the other groups, with corresponding changes of apoptosis-related proteins. Meanwhile, the *in vivo* tumor xenograft model showed that tumor inhibition rate in the PTX-Herceptin-MNPs-Fe_3_O_4_ group was higher than in the PTX-Herceptin group. Furthermore, PTX-Herceptin-MNPs-Fe_3_O_4_ enhanced the T2 imaging contrast enhancement effect on tumors in tumor-bearing mice.

**Conclusion:** The novel PTX-Herceptin-MNPs-Fe_3_O_4_ combination may represent a promising alternative breast cancer treatment strategy and may facilitate tumor imaging.

## Introduction

Breast cancer (BC) is one of the most common cancers, and the second leading cause of cancer-related mortality worldwide, representing a grievous threat to women's health and quality of life (1, 2). Human epidermal growth factor receptor-2 (HER-2) positive breast cancer accounts for 20–25% of all BC molecular subtypes. Its associations with aggressive tumor growth and inferior prognosis have been well-studied (3). HER-2 is a proto-oncogene that is negatively or minimally expressed in normal tissues and its overexpression could lead to excessive growth and enhanced invasiveness of tumor cells. HER-2-targeted agents effectively inhibit HER-2 expression, thereby achieving anti-tumor effect. Trastuzumab is a humanized anti-HER-2 monoclonal antibody that was first used in the therapy of HER-2 positive BC with changing its natural biology.

Paclitaxel (PTX) is clinically used as a chemotherapy agent in BC treatment. The efficacy of PTX combined with Herceptin for BC treatment is better than that of Herceptin alone. Several studies pointed out that BC cells exposed to PTX could produce HER2 receptor functional upregulation, making tumors more impressionable to the antiproliferative effects of Herceptin. The combination of Herceptin and PTX is recognized by the international medical community as an effective first-line treatment for patients with metastatic HER-2 positive BC (4). However, chemotherapeutic toxicity affects the application of chemotherapeutical drugs. Therefore, there has been an increasing demand for a more effective targeted drug delivery system addressing the issues of both chemotherapy resistance and toxicity, which has become a critical topic in cancer chemotherapy treatment.

Nanoparticles (NPs) can be used as carriers for transporting endocytosed drugs into tumor cells. Magnetic NPs (MNPs), in particular, offers the following advantages: First, it could offer targeted drug delivery by applying a magnetic field and connecting targeting ligands. Second, MNPs could overcome the limitations of traditional chemotherapy in terms of system distribution. Third, MNPs could generate synergistic effect with certain chemotherapy drugs through increasing the sensitivity of tumor tissues and the concentration of drugs in tumor cells (5, 6). With respect to magnetic nanoparticles Fe_3_O_4_ (MNPs-Fe_3_O_4_), it is considered to be one of the most promising nano biomaterials for its dual advantages of nanoparticles and magnetic property, such as MRI response to an external magnetic field (5). MNPs-Fe_3_O_4_ with superparamagnetism is a relatively simple preparation process and has an outstanding biocompatibility (6).

Interestingly, MNPs-Fe_3_O_4_ is able to accumulate at the tumor site under influence of the magnetic field after entering the human host, thereby causing embolization of tumor blood vessels to make tumor tissues ischemic and necrotic, and then promotes tumor sensitivity to anticancer drugs. Previous evidence demonstrated better outcomes and fewer side effects when MNPs-Fe_3_O_4_ served as carriers than traditional non-target drugs in treatment for lung, pancreatic, and hematological cancers (6, 7). We retrieved very few studies reporting the use of MNPs-Fe_3_O_4_ in HER-2positive BC therapies.

In this study, we synthesized a new type of nanoparticulate system whose surface was modified with Herceptin, and which consisted of biocompatible and biodegradable MNPs-Fe_3_O_4_. We prepared MNPs-Fe_3_O_4_ with superior crystallinity by high-temperature pyrolysis technology. Such high-performance was mainly reflected in size uniformity, regular morphology, higher magnetism and magnetocaloric effects, greater targeting ability, longer systemic circulation, and better biocompatibility. Our synthetic targeted MNPs are as follows. Oil-soluble iron oxide nanoparticles were synthesized using the high-temperature thermal decomposition technology. The surface of the nanoparticles was modified with DSPE-PEG-COOH to make it hydrophilic and the active functional group COOH was then loaded onto the surface. PTX and fat-soluble fluorescent dye Cy7 were loaded into the lipid layer. Herceptin monoclonal antibody (mAb) was attached to the surface of the nanoparticles. The targeted MNPs-Fe_3_O_4_ enhanced cellular drug absorption by cancer cells and increased their sensitivity to chemotherapy drugs, suggesting that the targeted MNPs-Fe_3_O_4_ might be an optimal nano drug delivery system (NDDS) for the clinical therapy of specific malignancies.

## Materials and Methods

### Main Materials and Apparatus

Iron (III) acetylacetonate [Fe(acac)3 (98%)] and OA (C17H33COOH, 85%) were purchased from Aladdin Chemical Reagent Co. Ltd. Benzyl ether (98%) was purchased from Alfa Aesar. DSPE-PEG2000 (PEG-phospholipids, 99%) was purchased from Shanghai A.V.T. Pharmaceutical Co., Ltd. Cy7 was purchased from Sangon Biotech (Shanghai) Co. Ltd. Paclitaxel (C_47_H_51_NO_14_, 98%) was purchased from Shanghai Yuanye Biotech Co., Ltd. Carbodiimide (EDC) and N-hydroxysulfosuccinimide sodium salt (sulfo-NHS) were purchased from Sigma-Aldrich (St Louis, MO). The commercially available reagents, including Chloroform (98%) and ethanol (95%), were all purchased from Sinopharm Chemical Reagent Co. Ltd. Monoclonal antibodies for cleaved-Caspase-3 and cleaved-PARP were supplied by Santa Cruz Biotechnology (Santa Cruz, CA, USA). All the chemicals were directly used without any further purification.

RPMI-1640 medium and trypsase were purchased from Gibco-Invitrogen Corp (Carlsbad, CA). 3-(4,5-dimethylthiazol-2-yl)-2,5-diphenyltetrazolium bromide (MTT) and dimethyl sulfoxide (DMSO) agents were obtained from Sigma-Aldrich (St Louis, MO). Other reagents included paclitaxel (Chemieliva Pharmaceutical Co. China) and Herceptin (Trastuzumab-Herceptin^®^, Genetech, San Francisco, CA, USA). High-performance liquid chromatography (HPLC) grade acetonitrile was purchased from Wanqing Chemical Reagent Co., Ltd. (Nanjing, People's Republic of China). Transmission electron microscope (TEM) was of a JEM-200CX model (JEOL Ltd., Akishima, Japan). The laser particle size analyzer and zeta potential analyzer were purchased from Malvern Instruments Ltd. (Worcestershire, UK). Flow cytometry was performed using a FACS Vantage SE model (Becton-Dickson, USA). Another apparatus was an optical microscope (OLYMPUS, CX31, Japan). All reagents used were of analytical grade.

### Preparation of MNPs-Fe_3_O_4_ (Fe_3_O_4_@OA)

The MNPs-Fe_3_O_4_ was synthesized and characterized by State Key Laboratory of Bioelectronics (Southeast University, People's Republic of China). MNPs-Fe_3_O_4_ was prepared by using iron acetylacetonate as the precursor of iron in the form of high temperature thermal decomposition. The selected reaction vessel was a three-neck flask with a capacity of 100 mL, the reaction solvent was dibenzyl ether, and the surfactants were oleic acid and oleylamine. In a specific experiment, the amount of iron acetylacetonate, dibenzyl ether and oleic acid was 2 mmol, 20 mL, and 3.8 mL, respectively. The reaction was placed in a three-necked flask. Nitrogen was blown into the mouth of the left bottle, circulating water condensed and refluxed at the mouth of the right bottle, and a temperature sensor placed in the middle bottle. After the system was successfully constructed, it was heated to 220°C at a heating rate of 3°C/min and held for 1 h. It was then further heated to 290°C at the same heating rate and reacted for 30 min to end the reaction. The heat source was removed with the reaction solution cooling down to room temperature before being poured into a beaker; the solution was then added with ethanol and magnetically separated. It was washed with ethanol three times, and kept in 10 mL of chloroform to a constant volume.

### MNPs-Fe_3_O_4_ Water Phase Transfer and Hydrophobic Molecular Loading (MNPs-Fe_3_O_4_@PEG)

The PEGylated long-circulating lipid DSPE-PEG2000 molecule was modified on the surface of MNPs-Fe_3_O_4_ to ensure its biocompatibility and water solubility, so that the active functional group COOH could be introduced onto the surface. In a specific experiment, 100 mg DSPE-MPEG2000 powder and 10 mg DSPE-PEG2000-COOH powder were weighed and dissolved in 3 mL of chloroform. Two mL of the above MNPs-Fe_3_O_4_ (concentration: 6 mg Fe/mL, dispersed in chloroform) were removed. The two were mixed into a 50 mL round bottom flask, and 10 mg of fluorescent dye cy7 and 10 mg of PTX were added simultaneously. It was fully sonicated with an ultrasound system for 5 min, before 3 mL of deionized water was added. It was continued to be sonicated for 3 min to form a milky turbid liquid. It was then rotated and evaporated at 70°C for 15 min to fully evaporate and remove the organic reagents. The sample was filtered through a 220 nm filter and stored in a refrigerator at 4°C.

### MNPs-Fe_3_O_4_ Surface Coupled With Herceptin mAb (MNPs-Fe_3_O_4_@PEG@mAb)

The 15 mg MNPs-Fe_3_O_4_@PEG aqueous solution was added to 180 mg of EDC solid powder and 200 mg of NHS solid powder, fully dissolved and shaken on a shaker for 30 min (120 r/min) to ensure -COOH on the nanoparticle surface activated. After activation, it was centrifuged by ultrafiltration and washed three times with deionized water to remove excess EDC and NHS. Next, the above sample was dispersed in 20 mL of borate buffer (0.02 mol/L, pH = 8.0) with a quantitative Herceptin dilution added dropwise, and incubated on a shaker at room temperature for 24 h (100 r/min). Finally, it was centrifuged by ultrafiltration and washed for three times with deionized water to obtain a black translucent Herceptin antibody-coupled magnetic nanoprobe solution, which was filtered through a 220 nm filter and stored at 4°C refrigerator.

### Determination of Drug Loading and Encapsulation Efficiencies

The drug loading and encapsulation efficiencies were determined using HPLC with an absorption peak at 230 nm, and were calculated according to the following equations (8):


$$\begin{array}{l} \text{ Loading efficiency }(\text{LE};\%)\text{ = }(\text{amount of drug in drug}-\text{loaded NPs/amount of drug}-\text{loaded NPs})\;\times \;\text{100}\%;\\ \text{Encapsulation efficiency }(\text{EE};\%)\text{ = }(\text{amount of drug in drug}-\text{loaded NPs/initial amount of drug})\;\times \;\text{100}\% \end{array}$$

### Characterization of Sample Physical and Chemical Properties

Transmission electron microscopy (TEM) was used to characterize morphologic size of MNPs-Fe_3_O_4_ and MNPs-Fe_3_O_4_@PEG, and dynamic light scattering (DLS) was used for magnetic nanoprobe MNPs-Fe_3_O_4_@PEG@mAb characterization of hydrodynamic dimensions. A vibrating sample magnetometer (VSM) was used to detect the saturation magnetization of MNPs-Fe_3_O_4_ to verify the properties of magnetic iron oxide nanoparticles.

### Microscopic Characteristics of Drug-Loaded NPs

The diameter of the NPs was between 1 and 100 nm, and the magnification of the general instrument was not enough to observe its microstructure. The resolution of TEM could reach 0.1–0.2 nm, which was an important instrument for studying NPs. The MNPs-Fe_3_O_4_ could be directly penetrated by the transmission electron beam when observed by TEM (7, 9). When the sample was prepared, it was only necessary to dilute the nanoparticles in an alcohol solution, and then the sample was picked up with a copper mesh with a carbon film, which was dried and observed with a TEM.

### Cell Line and Culture

The human breast cancer cell line (SK-BR-3 and MDA-MB-231 cells) was purchased from Shanghai Cell Bank of Chinese Academy of Sciences. SK-BR-3 and MDA-MB-231 breast cancer cells were adherent growth cells. In the experiment, RPMI1640 medium (containing 10% serum and 100 μl of double antibody) was used for cultivation. The incubation conditions were 37°C and 5% CO_2_. The cells were observed under a microscope. When the cell fusion rate reached 86% or more, SK-BR-3 and MDA-MB-231 breast cancer cells were subcultured using trypsin digestion solution.

### MTT Assay

Cell proliferation was measured by MTT assay *in vitro*. According to our preliminary experiments, inhibitory effects of the drugs were the most significant at 48 h (7). 5 × 10^3^ SK-BR-3 and MDA-MB-231 breast cancer cells in normal culture medium were seeded into each well of a 96-well plate. The cells were then cultured for 48 h and then washed and collected, following the manufacturer protocol, and then the optical density (OD) value was read at 570 nm using a microplate reader. The inhibition rate of cells was determined as follows: (1-OD of treated group/OD of control group) × 100%, and the cell viability was assessed as follows: OD of treated group/OD of control group × 100% (7).

### Flow Cytometric (FCM) Apoptosis Assay

SK-BR-3 and MDA-MB-231 breast cancer cells were seeded into six-well plates at the density of 4 × 10^5^ cells per well, treated as described in cell cycle analysis, and incubated at 37°C for 48 h. The washed cells were then suspended with 500 μL binding buffer and labeled with 5 μL Annexin V-FITC for 15 min at room temperature in dark. Thereafter, cell apoptosis was determined by FACSCalibur FCM (Becton-Dickinson, Franklin Lakes, NJ, USA) (7).

### Western Blot Analysis

After treatment, the total proteins were extracted from each group; protein concentration was measured using the Bradford method. Proteins were subjected to 10% sodium dodecyl sulfate–polyacrylamide gel electrophoresis (SDS-PAGE) and then transferred to a polyvinylidene difluoride membrane. The membrane was incubated with skimmed milk (5%) as a blocking agent for 1 h, followed by incubation with cleaved-PARP monoclonal antibodies overnight at 4°C. After washing, the membrane was incubated with peroxidase-labeled secondary antibody for 2 h at room temperature. The protein bands were visualized using the ECL system (Amersham, Buckinghamshire, UK) and analyzed using the Gel-Pro32 software (8).

### BC Xenograft Model in Nude Mice

BALB/c-nu mice (aged 6 weeks, weighted 18–22 g, and half male and half female) were purchased from Shanghai National Center for Laboratory Animals (Shanghai, People's Republic of China). They were maintained in specific pathogen-free conditions. Animal care, surgical procedures, and experimental protocols were approved by the Medical Ethics Committee on the Care and Use of Laboratory Animals of Shanghai Jiao Tong University.

The SK-BR-3 cells in the logarithmic growth phase were taken, and centrifuged (1,200 r/min) for 5 min. The cells were then resuspended with the cell density adjusted to 5 × 10^6^ cells/mL. 200 μl of the cell suspension was used to subcutaneously inoculate cells on the right hindlegs of mice and the injected site developed grain-sized tumors. When the tumor size reached 75–150 mm^3^, a total of 36 nude mice were randomly divided into six groups: Group A, control group, mice were treated with 0.2 mL 0.9% normal saline; Group B, MNPs-Fe_3_O_4_; Group C, PTX; Group D, PTX-MNPs-Fe_3_O_4_; Group E, PTX and Herceptin; Group F, PTX-Herceptin-MNPs-Fe_3_O_4_. These nude mice were injected intravenously with the respective treatment every other day. Based on the drug loading efficiency (7, 10), PTX concentration was 1 mg/kg and Herceptin concentration was 300 ug/kg.

### Tumor Growth Measurement and Inhibition Rate

Toxicity symptoms were monitored throughout the entire study period. Length (a) and width (b) of tumors were measured using a digital caliper and tumor volume (V/mm^3^) was calculated according to the formula V = 1/2 (a × b^2^), where a and b represented the longest and shortest diameter, respectively (9). Change in tumor size among different groups was recorded as the relative tumor volume (RTV) using the formula RTV = V_X_/V_1_, where V_X_ and V_1_ represented the volumes on day X and the first day of tumor treatment, respectively. Antitumor inhibition rate was defined as the inhibitory rate, calculated using the formula inhibitory rate (IR) (%): (1-average experimental group RTV/average control group RTV) × 100%. All studies were performed in adherence to the Guidelines for the Care and Use of Laboratory Animals established by the National Institute of Health (11).

### Immunohistochemistry Analysis

Tumor tissue was cut into 5 μm slices, and expression of cleaved-Caspase-3 protein was detected by an immunohistochemical staining SP method. The sections were incubated with anti-cleaved-Caspase 3 antibody (working dilution 1:100) at 4°C overnight. After washing, the sections were reintubated with a secondary anti-mouse biotinylated antibody (1:1,000) in a dark room for 1 h. Positive cells were counted within five randomly selected horizons for each slice at a magnification of 400 × (8, 11).

### *In vivo* Magnetic Resonance

The German Bruker 7.0T Micro-MR imaging system was used to detect magnetic resonance imaging of tumors in tumor-bearing mice. The inner diameter of the horizontal scanning frame was 10 cm and the mouse coil was used. The mice were anesthetized with 5% isoflurane and placed onto a plexiglass scanning bed. The head of the mouse was fixed. The anesthesia state of the mice was maintained with 1.5% isoflurane air mixed gas. The respiratory rate was adjusted to 40–60 times/min. PTX-Herceptin-MNPs-Fe_3_O_4_ was injected into the tail vein before (0 h), and 8 h after injection to perform magnetic resonance scanning imaging. The magnetic resonance imaging sequence and parameters were as follows: FLASH-multi-slice T2 ^*^ (Fast Low Angle Shot, FLASH) sequence: repetition time TR = 333.1 ms, echo time TE = 5.0 ms, flip angle (Flip Angle): 30.0°, 1 repetition time, Field of view (FOV): 4 cm × 4 cm, acquisition matrix 512 × 512, layer thickness 1 mm, interval 0–0.1 mm.

### *In vivo* Fluorescence Imaging

Tumor-bearing mice were treated with local hair removal and cleaned with warm water to minimize the non-specific fluorescence effects on relevant areas. The mice were placed in a gas anesthesia system for isoflurane anesthesia and scanned, followed by PTX-Herceptin-MNPs-Fe_3_O_4_ injection through the tail vein at 0–8 h for whole-body near-infrared fluorescence distribution imaging. The optical imaging parameters were as follows: excitation wavelength 704 nm, NIR emission filter.

### Statistical Analysis

Quantitative data were described as means ± standard deviations (SDs). Intergroup differences were analyzed by *F*-test. The threshold for significance was *P* = 0.05. All statistical analyses were conducted using SPSS, Version 15.0 (SPSS Inc., Chicago, IL, USA).

## Results

### Drug Loading and Encapsulation Efficiencies

The drug loading efficiencies were 5.5% ± 0.2% for PTX and 3.1% ± 0.1% for Herceptin, and the encapsulation efficiencices were 84.5% ± 2.3% for PTX and 77.6% ± 1.9% for Herceptin, respectively. The molar ratio of PTX to Herceptin was ~2:1 in the NPs, which was an appropriate proportion for targeted efficacy. There were no significant differences in drug loading and encapsulation efficiencies between the repeated batches of MNPs-Fe_3_O_4_. These results further proved that the formulation process was stable and that the MNPs-Fe_3_O_4_ could be an effective drug delivery carrier.

### Characteristics of MNPs-Fe_3_O_4_

**Figure 1:**
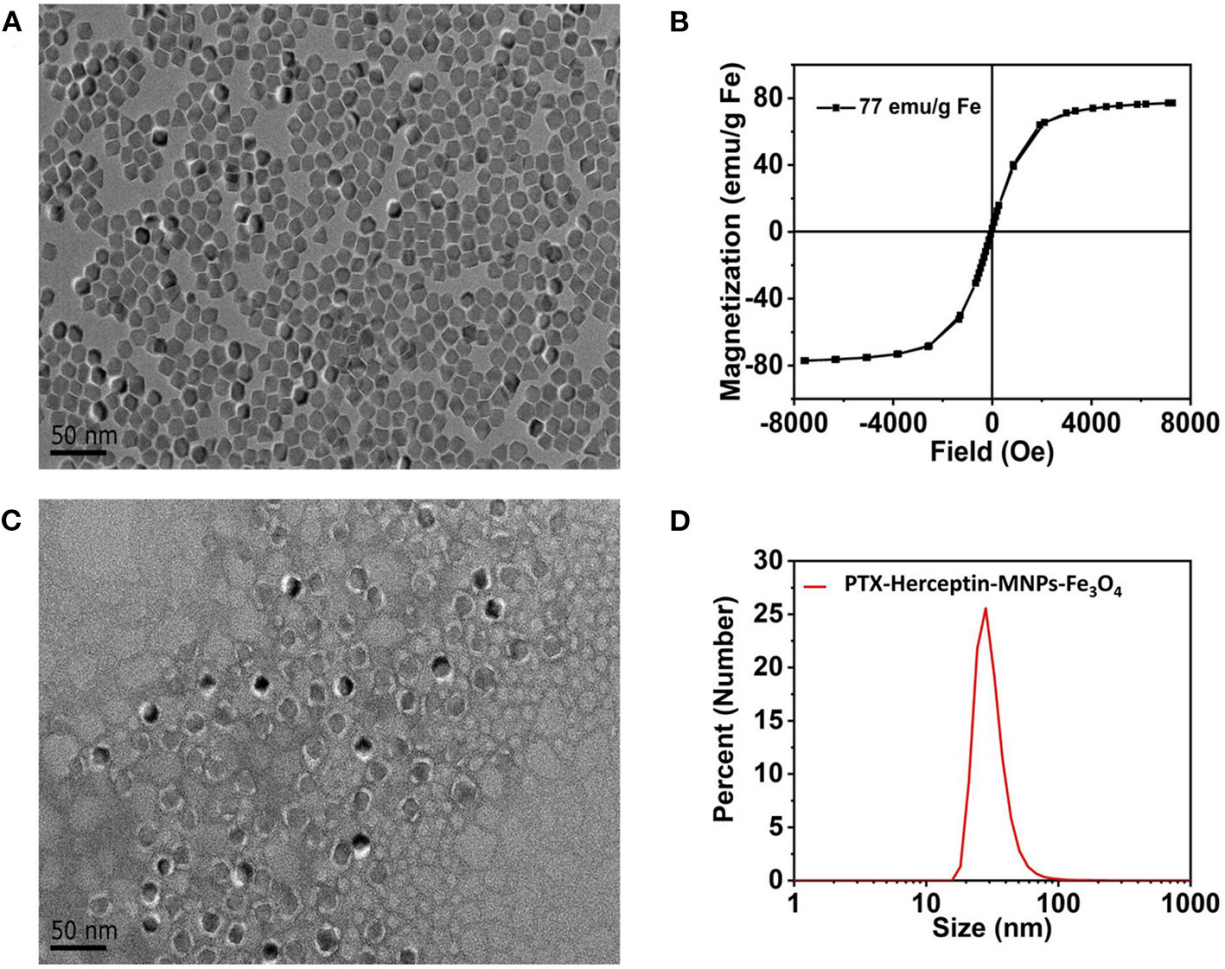
**(A)** TEM image of Fe_3_O_4_@OA nanoparticles. **(B)** Hysteresis loops of Fe_3_O_4_@OA nanoparticles measured at 300 K. **(C)** TEM image of PTX-Herceptin-MNPs-Fe_3_O_4_. **(D)** DLS size measurement of PTX-Herceptin-MNPs-Fe_3_O_4_.

Figure 1A displays the findings of the TEM analysis of Fe_3_O_4_@OA nanoparticles from which we found that most nanoparticles were spherical in shape and uniform in 10-nanometer size. As shown in Figure 1B, the saturation magnetization of Fe_3_O_4_@OA was 77 emu/g while the coercivity and remanence were zero, indicating that nanoparticles had strong magnetic and superparamagnetic properties.

According to the TEM image (Figure 1C) of negatively stained PTX-Herceptin -MNPs-Fe_3_O_4_ with 2% phosphotungstic acid, all samples acquired a typical monodisperse core-shell structure (the clear white circles of outer layers). In addition to the core of the magnetic particle, the outer layers of this structure were mainly composed of phospholipid molecules (2 nm). The fluorescent molecules Cy7 and the drug PTX were inserted into the white lipid layer on the surface of the magnetic particles, indicating that the nanoparticles had significant fluorescence properties and drug loading capacities. The DLS results (Figure 1D) identified that the hydrodynamic size of PTX-Herceptin-MNPs-Fe_3_O_4_ was 28 nm.

### MNPs-Fe_3_O_4_ Enhanced the Proliferation-Inhibiting and Cytotoxicity Effects of PTX and Herceptin *in vitro*

**Figure 2:**
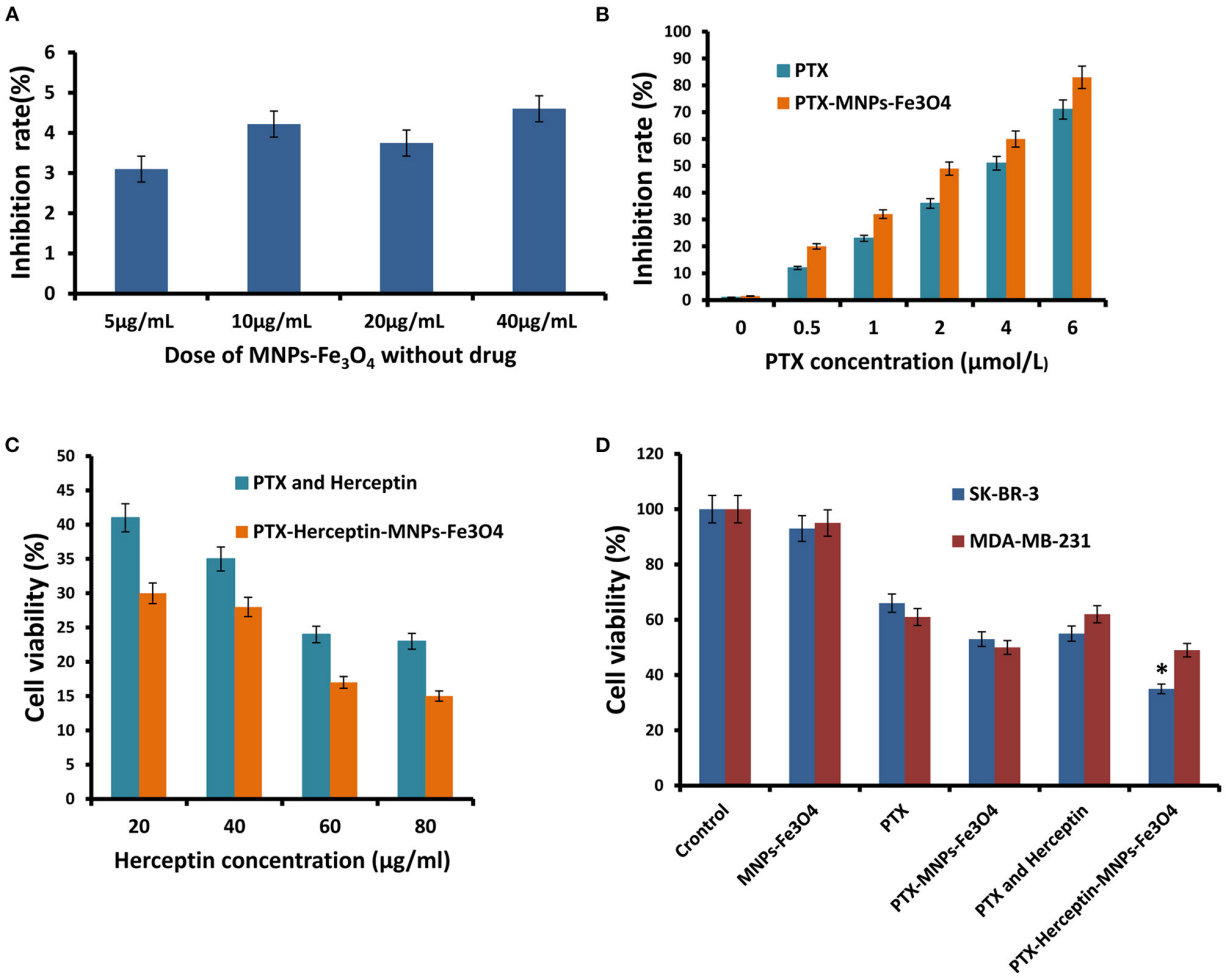
**(A)** Inhibitory effects of the different concentrations of MNPs-Fe_3_O_4_ on the growth of SK-BR-3 cells using the MTT assay. **(B)** Inhibitory effects of the combination of 20 μg/mL MNPs-Fe_3_O_4_ with different concentrations of PTX on the growth of SK-BR-3 cells. **(C)** Viability of SK-BR-3 cells treated with different concentrations of Herceptin. **(D)** Viability of SK-BR-3 and MDA-MB-231 cells treated with control, MNPs-Fe_3_O_4_, PTX, PTX-MNPs-Fe_3_O_4_, PTX-Herceptin, and PTX-Herceptin-MNPs-Fe_3_O_4_. ^*^P < 0.05 when compared with the PTX and Herceptin group.

The dose-effect curve for the inhibition rate when SK-BR-3 cells were exposed to MNPs-Fe_3_O_4_ (5–40 μg/mL) for 48 h was shown in Figure 2A (7), and MNPs-Fe_3_O_4_ with concentrations of <40 μg/mL had no obvious effect on proliferation (*P* > 0.05). The anticancer efficacy of nanoparticles is reflected by their cytotoxicity effect. PTX enhanced the rate of inhibition of SK-BR-3 cells in a dose-dependent manner; furthermore, PTX-MNPs-Fe_3_O_4_ increased the cytotoxicity effect on SK-BR-3 cells compared with PTX (Figure 2B).

SK-BR-3 cells have positive HER2 expression, and MDA-MB-231 cells belong to the basal-like subtype due to the negative estrogen receptor (ER), progesterone receptor (PR), and HER2 expressions (10). SK-BR-3 and MDA-MB-231 breast cancer cells were used to detect the targeting effect. We evaluated the combinations of PTX-MNPs-Fe_3_O_4_ with different concentrations of Herceptin to find the optimal combination targeting SK-BR-3 cells. MTT assays using the SK-BR-3 and MDA-MB-231 cell lines were performed to analyze the potential cytotoxicity of PTX-Herceptin-MNPs-Fe_3_O_4_. As shown in Figure 2C, the SK-BR-3 cell viability after PTX-Herceptin-MNPs-Fe_3_O_4_ treatment was negatively correlated with the concentration of Herceptin. As shown in Figure 2D, the PTX-Herceptin-MNPs-Fe_3_O_4_ exhibited the strongest cytotoxicity among all the groups (control group, MNPs-Fe_3_O_4_ group, PTX group, PTX-MNPs-Fe_3_O_4_ group, PTX-Herceptin group, and PTX-Herceptin-MNPs-Fe_3_O_4_ group) using the SK-BR-3 cell line (*P* < 0.05). However, there was no significant difference in the viability of MDA-MB-231 cells between the PTX-MNPs-Fe_3_O_4_ and PTX-Herceptin-MNPs-Fe_3_O_4_ groups (*P* > 0.05).

### MNPs-Fe_3_O_4_ Enhanced the Apoptosis-Promoting Effects of PTX and Herceptin *in vitro*

**Figure 3:**
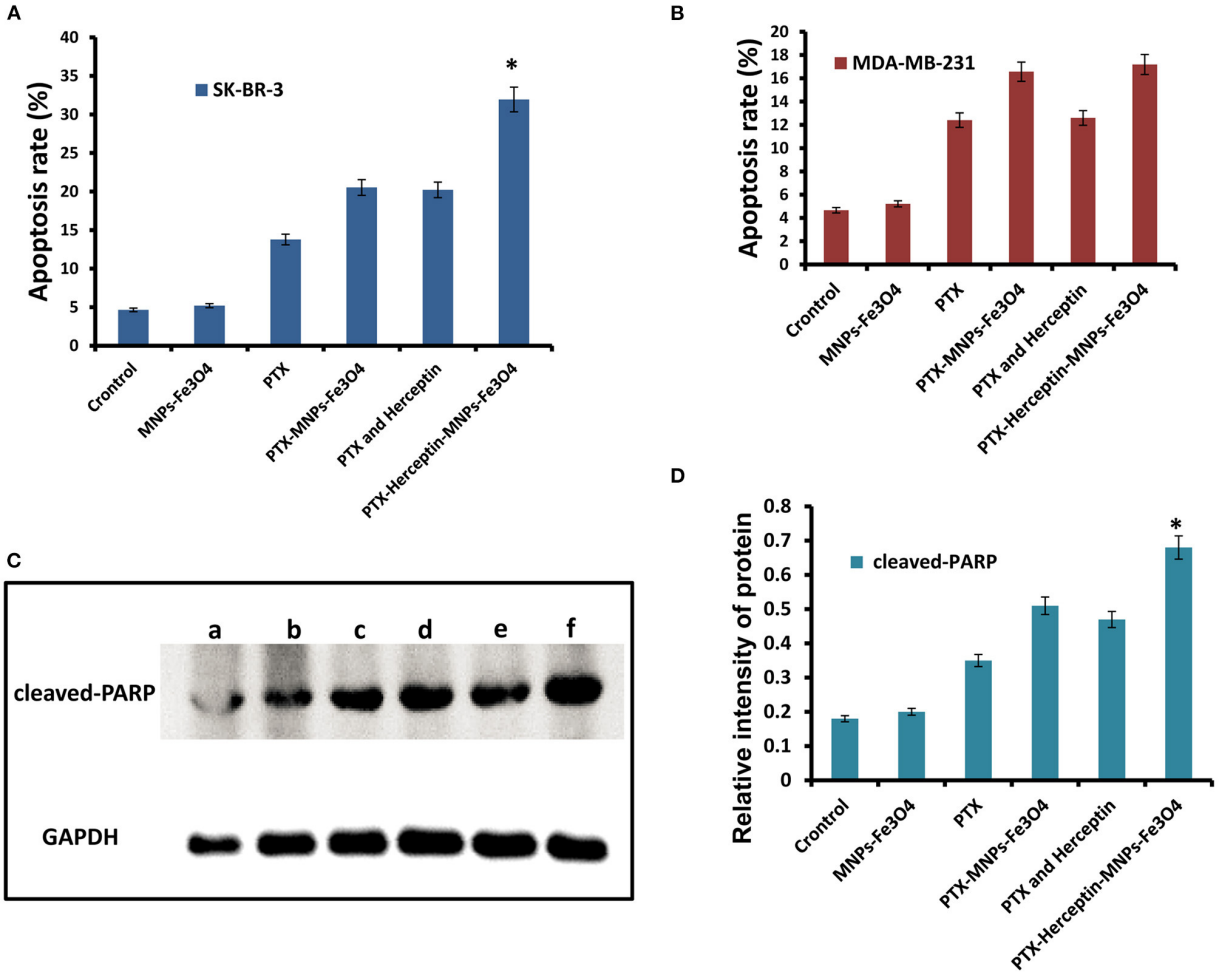
**(A)** Apoptosis rate of SK-BR-3 cells in different groups. **(B)** Apoptosis rate of MDA-MB-231 cells in different groups. **(C)** Levels of cleaved-PARP (a, Control group; b, MNPs-Fe_3_O_4_ group; c, PTX group; d, PTX-MNPs-Fe_3_O_4_ group; e, PTX and Herceptin group; and f, PTX-Herceptin-MNPs-Fe_3_O_4_ group) and **(D)** relative intensity of cleaved-PARP in SK-BR-3 cells after treatment in different groups. ^*^P < 0.05 when compared with the PTX and Herceptin group.

Figure 3A presents no significant difference in apoptosis rate between SK-BR-3 cells treated with MNPs-Fe_3_O_4_ (4.66% ± 0.51%) and the control group (5.21% ± 0.82%) (*P* > 0.05). The apoptosis rates of SK-BR-3 cells treated with PTX and PTX-MNPs-Fe_3_O_4_ were 13.78% ± 0.33% and 20.53% ± 0.42%, respectively. Besides, the rate was 20.22% ± 0.27% for SK-BR-3 cells induced by 0.5 μmol/L PTX-Herceptin, and 31.95% ± 0.64% (*P* < 0.05) for cells treated by PTX-Herceptin-MNPs-Fe_3_O_4_, respectively. Among the different treatments (Figure 3B), there was no significant difference in the apoptosis rate of MDA-MB-231 cells between the PTX-MNPs-Fe_3_O_4_ (16.57% ± 1.28%) and PTX-Herceptin-MNPs-Fe_3_O_4_ groups (17.19% ± 0.94%) (*P* > 0.05). The results indicated that PTX-Herceptin-MNPs-Fe_3_O_4_ had no targeted effect on MDA-MB-231 cells with no expression of HER2 antigen.

As shown in Figure 3C, in comparison with the control group, the levels of cleaved PARP were higher in the PTX, PTX-MNPs-Fe_3_O_4_, PTX-Herceptin, and PTX-Herceptin-MNPs-Fe_3_O_4_ groups. The level of cleaved PARP was markedly higher in the PTX-Herceptin-MNPs-Fe_3_O_4_ group than in the other groups (*P* < 0.05; Figure 3D).

### MNPs-Fe_3_O_4_ Increased the Tumor Inhibition Capability of PTX and Herceptin *in vivo*

**Figure 4:**
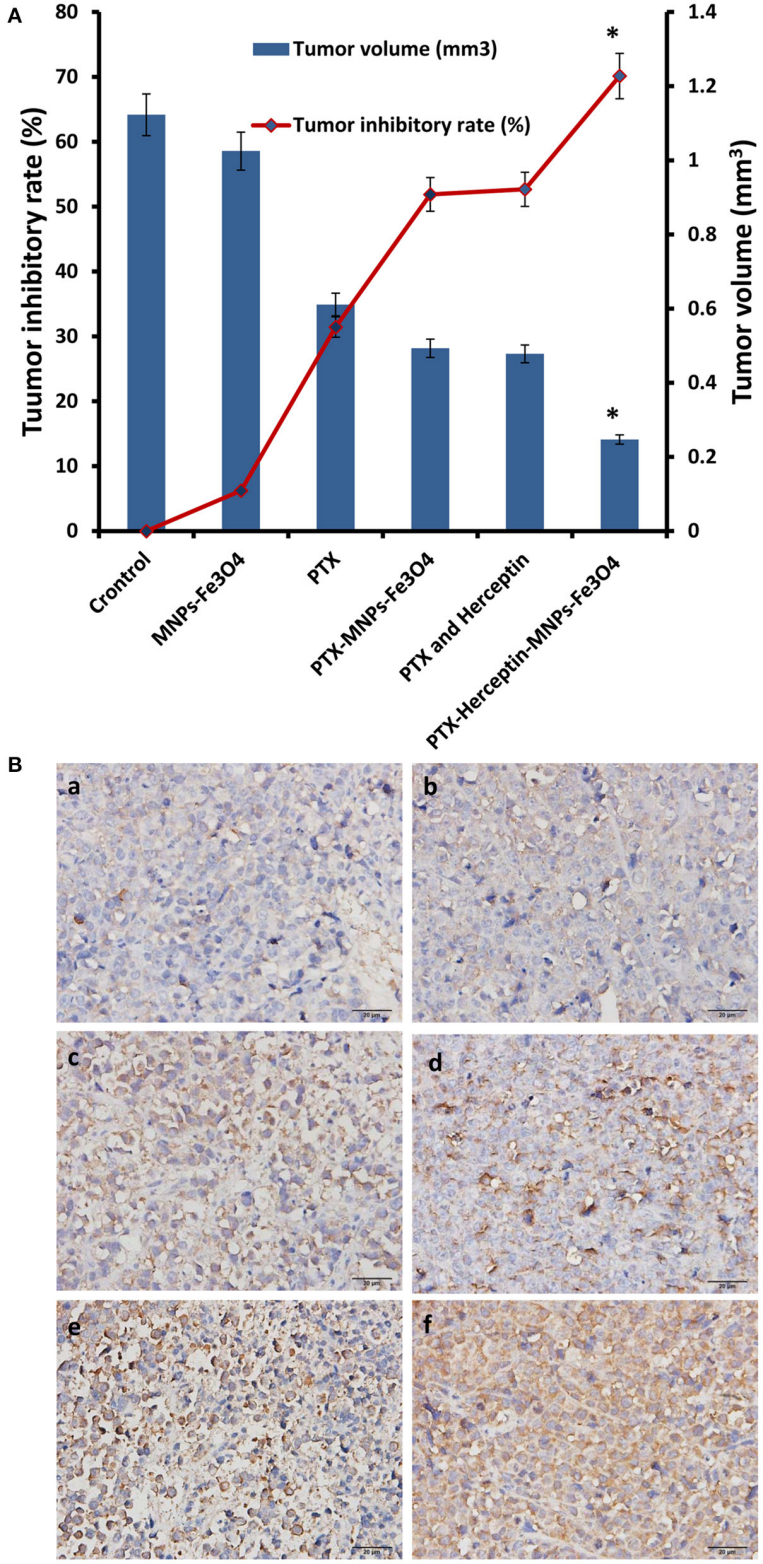
**(A)** The relative tumor inhibitory rate of mice after treatment for 14 days. **(B)** Expression of cleaved-Caspase-3 in tumor tissues after the treatment (immunohistochemistry, × 400) of (a) Control group; (b) MNPs-Fe_3_O_4_ group; (c) PTX group; (d) PTX-MNPs-Fe_3_O_4_ group; (e) PTX and Herceptin group; and (f) PTX-Herceptin-MNPs-Fe_3_O_4_ group. ^*^P < 0.05 when compared with the PTX and Herceptin group.

All mice were injected via tail veins for 14 days and no obvious symptoms of toxicity were observed during the treatment. As shown in Figure 4A, there was no obvious difference in the inhibitory rate between the control group and the MNPs-Fe_3_O_4_ group (*P* > 0.05) after treatment. The inhibition rates in the PTX group, PTX-MNPs-Fe_3_O_4_ group, PTX-Herceptin group, and PTX-Herceptin-MNPs-Fe_3_O_4_ group were 31.46% ± 2.67%, 51.89% ± 3.58%, 52.68% ± 4.12%, and 70.14% ± 3.63%, respectively. The tumor inhibition rate of the PTX-Herceptin-MNPs-Fe_3_O_4_ group (70.14% ± 3.63%) was significantly higher than the PTX-Herceptin group (52.68% ± 4.12%) (*P* < 0.05). These results suggested that PTX-Herceptin-MNPs-Fe_3_O_4_ had the strongest effect of tumor inhibition on SK-BR-3 xenografts.

### MNPs-Fe_3_O_4_ Enhanced the Apoptosis-Promoting Effects of PTX and Herceptin *in vivo*

Immunohistochemistry was used to detect the expression of cleaved-Caspase-3 in the tumor tissues of nude mice (Figure 4B). Immunohistochemical staining for positive expression was shown as fine brown particles, which were mainly located on the cell membrane and in the cytoplasm of tumor cells, and which exhibited diffuse or focal distribution. The PTX-Herceptin-MNPs-Fe_3_O_4_ group showed the strongest cleaved-Caspase-3 expression. Compared with the other two groups (control group and MNPs-Fe_3_O_4_ group), the levels of cleaved-Caspase-3 in the PTX group, PTX-MNPs-Fe_3_O_4_ group, PTX-Herceptin group, and PTX-Herceptin-MNPs-Fe_3_O_4_ group were higher with more staining-positive cells. However, there was no significant difference between the control group and the MNPs-Fe_3_O_4_ group.

### PTX-Herceptin-MNPs-Fe_3_O_4_ had T2 Imaging Contrast Enhancement Effect on Tumor *in vivo*

We selected mice subcutaneously inoculated with tumors as animal models to examine the *in vivo* effects of PTX-Herceptin-MNPs-Fe_3_O_4_ (300 μg Herceptin/kg). We loaded the fluorescent component Cy7 into the hydrophobic inner layers of PEG-modified magnetic nanoprobe, and achieved a dual-modal nanostructure with both optical and magnetic properties. Therefore, we used the near-infrared fluorescence and magnetic resonance imaging (MRI) technology to monitor the enrichment of magnetic nanoprobes in mouse tumors both intuitively and *in situ*.

**Figure 5:**
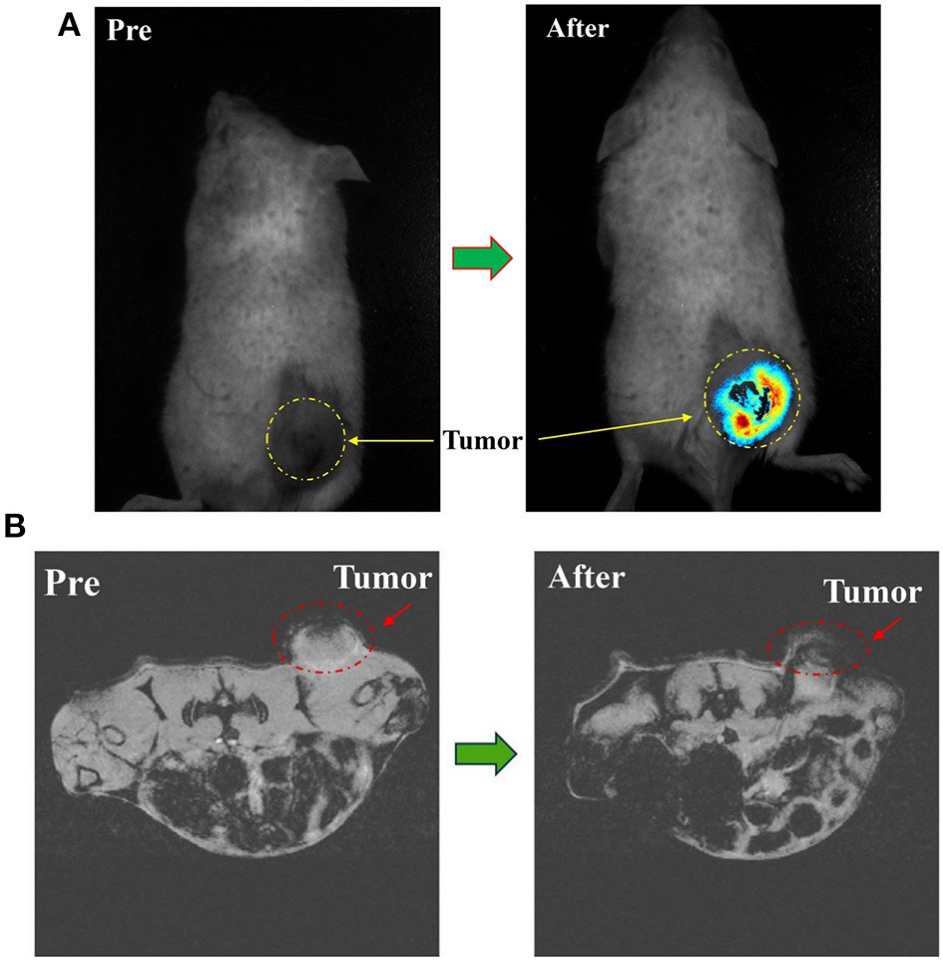
**(A)** *In vivo* NIRF imaging of tumor mice before and 8 h after tail vein injection of PTX-Herceptin-MNPs-Fe_3_O_4_. The yellow dashed circles indicate the tumor. **(B)** T^*^2-Weighted MR images of the mice tumor before and 8 h after tail vein injection of PTX-Herceptin-MNPs-Fe_3_O_4_ using a 7 T MR scanner. The red dotted circles displayed the tumor site.

During the systemic circulation, magnetic nanoprobe was partially engulfed in liver and spleen due to the unique physiological structure of the tumor tissue (i.e., the permeability and retention (EPR) effect), as well as the surface of the magnetic nanoprobe modified by the PEG molecule with anti-RES phagocytosis and tumor-targeted Herceptin antibody. Another part of the nanoprobe was selectively distributed in tumor tissue and had a long-term enrichment. Fluorescence intensity at the tumor site was greatly enhanced as shown in Figure 5A.

From the MRI images (Figure 5B), the tumor became darker while the signal value of the tumor site reached a peak at 8 h after PTX-Herceptin-MNPs-Fe_3_O_4_ injection, which proved that PTX-Herceptin-MNPs-Fe_3_O_4_ had T2 imaging contrast enhancement effect on tumor in tumor-bearing mice. Also, we found that PTX-Herceptin-MNPs-Fe_3_O_4_ had a considerable amount of enrichment in tumors, laying a foundation for the subsequent treatment with release of anticancer drugs.

## Discussion

Nanoparticles have developed rapidly for cancer treatment as they overcome the limitations of conventional small molecule chemotherapy drugs. Relying on the nano drug carrier system, a meaningful approach should include reducing systemic dose, improving local drug concentration in tumor tissues, and achieving targeted drug delivery (12). As nanomedicine carriers, magnetic nanoparticles have come into focus currently because of their potential on multi-targeting (13, 14). First, MNPs could conduct passive targeting by enhanced EPR, while targeting actively by magnetic field effect or surface modification of targeting ligands, thus play its role effectively on antitumor activities (15). Moreover, with the characteristic of tumor cells targeted, MNPs are able to help with the reduction of drug dosage. This is exemplified by the combination of MNPs and paclitaxel, methotrexate, mitoxantrone, and adriamycin, which improve the target specificity (16). Such nanocomposites with magnetic and pH dual-responsive performance provided an outstanding platform for enhanced drug-resistant BC treatment, and achieved not only chemotherapy photodynamic therapy in tumor treatments, but controlled drug release and alleviated side effects (17). Rejinold et al. synthesized MNPs-Fe_3_O_4_ with non-invasive radiofrequency (RF) to prolong its circulation time in 4T1 breast cancer cells and enhance apoptosis effects (18).

Compared with other magnetic nanomaterials, MNPs-Fe_3_O_4_ is more stable with higher hardness. Because of its strong magnetism, as well as simple preparation and good biocompatibility, Fe_3_O_4_ is potentially for magnetically guided drug delivery. Our previous study has proved that Fe_3_O_4_ remains stable within the water phase and therefore is able to elongate blood circulation without been rapidly metabolized (7). What we know about MNPs-Fe_3_O_4_ is largely based upon studies investigating its use as drug carriers and synergistic chemotherapy drugs against leukemia resistance (6, 13). It has been shown *in vivo* and *in vitro* and in clinical experiments that MNPs-Fe_3_O_4_ has lower toxicity, and better effects in reversing the resistance of synergistic chemotherapy drugs.

**Figure 6:**
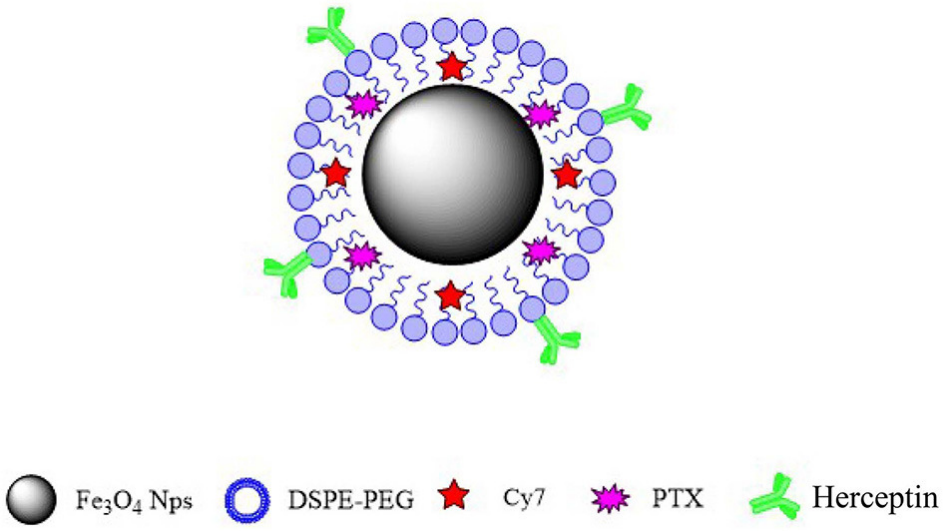
Schematic representation of PTX-Herceptin-MNPs-Fe_3_O_4_.

In this study, we innovatively synthesized a new type of nanoparticle system (Figure 6), whose surface was modified with Herceptin, and which consisted of biocompatible and biodegradable magnetic nanoparticles (MNPs)-Fe_3_O_4_. We used MNPs-Fe_3_O_4_ as the NDDS to demonstrate the potential targeted effects in anticancer therapy. First, results of the MTT assay confirmed a good biocompatibility of MNPs-Fe_3_O_4_. We explored the combinatorial chemotherapeutic efficacy of MNPs-Fe_3_O_4_ with PTX-Herceptin on BC *in vitro* (using SK-BR-3 and MDA-MB-231 cell lines). It was observed that the viability of SK-BR-3 cells decreased with higher concentrations of PTX-MNPs-Fe_3_O_4_. We also compared the cytotoxicity of PTX-Herceptin-MNPs-Fe_3_O_4_ to SK-BR-3 and MDA-MB-231 cells. PTX-Herceptin-MNPs-Fe_3_O_4_ could specifically recognize HER2 antigen on SK-BR-3 cells, and showed greater cytotoxicity to SK-BR-3 cells than MDA-MB-231 cells. Our observations indicated that PTX-Herceptin-MNPs-Fe_3_O_4_ had the ability to bind to SK-BR-3 cells, and promoted the targeted antitumor effect on HER2-positive breast cancer cells. In addition, the apoptotic rate of SK-BR-3 cells treated with PTX-Herceptin-MNPs-Fe_3_O_4_ increased significantly compared with those of cells treated with PTX, PTX-MNPs-Fe_3_O_4_, PTX, and Herceptin. PTX-Herceptin-MNPs-Fe_3_O_4_ dramatically increased the cleaved-PARP levels in SK-BR-3 cells, which supported the promotion of PTX-Herceptin-induced apoptosis by MNPs-Fe_3_O_4_. Furthermore, it was proved that the tumor size was better controlled in PTX-Herceptin-MNPs-Fe_3_O_4_ group than in the PTX group, PTX-MNPs-Fe_3_O_4_ group, and PTX-Herceptin group. Third, the expression of cleaved-Caspase 3 indicated that the targeted PTX-Herceptin-MNPs-Fe_3_O_4_ DDS substantially boosted antitumor activity of traditional chemotherapeutic agents. Last, in the xenograft model, there apparently appeared enrichment in tumor tissue site with the strongest fluorescence in tumor-bearing mice under the action of external magnetic field, 8 h after PTX-Herceptin-MNPs-Fe_3_O_4_ was injected, which verified the property of magnetic targeting. The *in vivo* tumor xenograft model showed that the tumor inhibition rate in the PTX-Herceptin-MNPs-Fe3O4 group was higher than in the PTX-Herceptin group. Furthermore, PTX-Herceptin-MNPs-Fe_3_O_4_ enhanced the T2 imaging contrast enhancement effect on tumors in tumor-bearing mice. These findings suggest that the novel PTX-Herceptin-MNPs-Fe_3_O_4_ combination may represent a promising alternative breast cancer treatment strategy and may facilitate tumor imaging. Together these results provided important insights that targeted antitumor activity could be achieved with PTX-Herceptin-MNPs-Fe_3_O_4_ in our new delivery system.

Developments of nanomaterials have made a great breakthrough in diagnosis and treatment of tumors, especially in drug delivery system. Because of its role on tumor targeting, nanoparticles generate EPR under the tumor microenvironment of vascular anomalies and poor lymph drainage, and further overcome multidrug resistance (MDR) through increasing drug concentration in cancer cells and inhibiting tumor progression. Future studies should focus on magnetic nanoparticles and MDR.

## Conclusion

A new targeted nano drug delivery system is constructed using MNPs-Fe_3_O_4_ combined with Herceptin and the anti-tumor drug PTX. It effectively delivers anti-tumor drugs to breast cancer cells, and significantly improves the efficacy of chemotherapy. Our findings provide an innovative theoretical basis for clinical application by reducing dose and side effects of chemotherapy drugs on the basis of nanotechnology.

## Data Availability Statement

The raw data supporting the conclusions of this article will be made available by the authors, without undue reservation.

## Ethics Statement

The animal study was reviewed and approved by the Medical Ethics Committee on the Care and Use of Laboratory Animals of Shanghai Jiao Tong University.

## Author Contributions

LG, LH, YS, and JZ: conception or design. LG, HZ, PL, TM, DH, LS, LH, YS, and JZ: acquisition, analysis, or interpretation of data. LG: drafting of the manuscript and statistical analysis. HZ, PL, TM, DH, LS, LH, YS, and JZ: critical revision of the manuscript for important intellectual content. YS and JZ: administrative, technical, or material support. All authors contributed to the article and approved the submitted version.

## Conflict of Interest

TM, DH, and LS were employed by the company Lianren Digital Health Technology Company, LTD., Shanghai, China. The remaining authors declare that the research was conducted in the absence of any commercial or financial relationships that could be construed as a potential conflict of interest.

## Publisher's Note

All claims expressed in this article are solely those of the authors and do not necessarily represent those of their affiliated organizations, or those of the publisher, the editors and the reviewers. Any product that may be evaluated in this article, or claim that may be made by its manufacturer, is not guaranteed or endorsed by the publisher.
