# Supplementary material for: Preclinical Assessment of Paclitaxel- and Trastuzumab-Delivering Magnetic Nanoparticles Fe3O4 for Treatment and Imaging of HER2-Positive Breast Cancer
Source: Front Med (Lausanne). 2021 Oct 28;8:738775. doi: 10.3389/fmed.2021.738775 (PMC8581045; doi:10.3389/fmed.2021.738775)
Supplement: Supplementary file 4 [file Data_Sheet_2.docx]

Fe_3_O_4_ NPs





Fe_3_O_4_ based nanoprobe
