# Supplementary figures and images for: Preclinical Assessment of Paclitaxel- and Trastuzumab-Delivering Magnetic Nanoparticles Fe3O4 for Treatment and Imaging of HER2-Positive Breast Cancer
Source: Front Med (Lausanne). 2021 Oct 28;8:738775. doi: 10.3389/fmed.2021.738775 (PMC8581045; doi:10.3389/fmed.2021.738775)

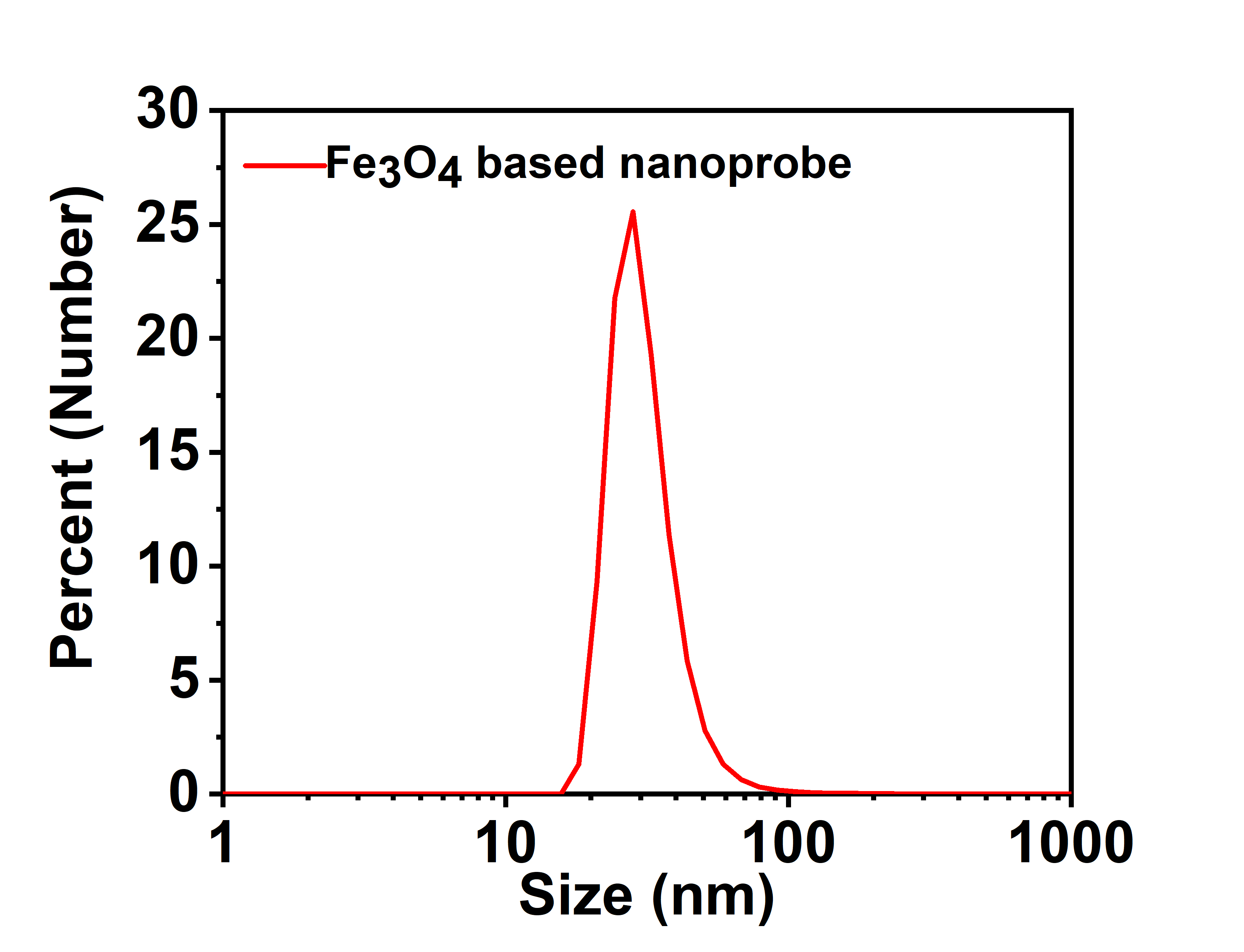

Supplement: Supplementary file 7 [file Image_2.JPEG]
